# Synergistic Effects of Non-Ionizing Radiation in the Targeted Modification of Living Tissues

**DOI:** 10.3390/ijms262311415

**Published:** 2025-11-26

**Authors:** Ilya Klabukov, Daria Eygel, Elena Isaeva, Anastas Kisel, Evgeny I. Isaev, Mikhail Potievskiy, Dmitrii Atiakshin, Victoria Shestakova, Denis Baranovskii, Bagavdin Akhmedov, Yana Sulina, Elizabeth Skornyakova, Peter Shegay, Andrey D. Kaprin

**Affiliations:** 1 National Medical Research Radiological Center, Koroleva Street 4, 249036 Obninsk, Russia; 2 Obninsk Institute for Nuclear Power Engineering, National Research Nuclear University MEPhI, Studgorodok 1, 249036 Obninsk, Russia; 3 Scientific and Educational Resource Center for Innovative Technologies of Immunophenotyping, Digital Spatial Profiling and Ultrastructural Analysis, Patrice Lumumba Peoples’ Friendship University of Russia (RUDN University), 117198 Moscow, Russia; 4 University Hospital Basel, Basel University, 4001 Basel, Switzerland; 5 Institute of Natural Science, Tsiolkovsky Kaluga State University, Stepana Razina Street 26, 248023 Kaluga, Russia; 6 Vishnevsky National Medical Research Centre of Surgery, 117997 Moscow, Russia; 7 Department of Obstetrics and Gynecology, Sechenov First Moscow State Medical University (Sechenov University), 119435 Moscow, Russia; 8 Department of Engineering, Russian University of Transport (MIIT), Obraztsova Street, 9, 127994 Moscow, Russia

**Keywords:** biophotonics, bosons, extracellular matrix, laser radiation, mechanotransduction, non-ionizing radiation, oncomatrix, photonics, physiological relevance, regenerative medicine, tissue engineering

## Abstract

Non-ionizing radiation and excited states can modify the properties of biological tissues, altering their structure, surface morphology and mechanical properties of the extracellular matrix, and stimulating resident cells. The primary goal of non-ionizing radiation applications is to achieve high precision and controllability in the processes of modifying biological tissues, allowing for the minimization of damage to surrounding healthy tissues and improving repair processes. The use of the photonic and acoustic methods can contribute to the creation of new materials with specific biological properties, which is particularly important for the development of individualized implants, efficient drug delivery systems, and tissue engineering methods. An important aspect is the development of integrated approaches that combine different types of non-ionizing radiation to achieve a synergistic effect we term a “bosonic concentrate.” For example, the combination of photonic and phononic ultrasonic therapy can improve the penetration of drugs into deeper tissue layers, while the combination of photothermal and acoustic exposure can increase the precision and efficiency of tumor cell removal. This review discusses the effects underlying the potential treatment options for biological tissue modification to improve their physiological relevance based on various bosonic concentrate combinations. In particular, we will discuss how low-energy acoustic phonons (characteristic energy 0.03–0.1 eV) could create tissue-specific spatially resolved structures that serve as matrices for optical photons (1–3 eV) and excitons (~0.1 eV) and how they could be focused and dissipated to mediate biochemical reactions. All of them are capable of propagating in living tissues, mediating changes at the cellular and molecular levels.

## 1. Introduction

The use of physical fields is an effective method for modifying the internal properties of various materials, including their physical, mechanical, and biochemical characteristics [1]. These changes caused by both the ionizing and non-ionizing irradiation could modify the biological properties of living tissues through specific cellular mechanoreception and mechanotransduction mechanisms [2,3,4,5]. In particular, changes in the micromechanical properties of the extracellular matrix caused by ionizing irradiation are primarily due to alterations in collagen fibers. Alterations in collagen organization are relatively minor compared to changes in chemical composition and lead to a disorganized secondary structure [6].

Low doses of ionizing irradiation do not lead to the visible modification of the tissue properties but can induce subtle detrimental changes, such as disorganization of the secondary structure of collagen, which compromises micromechanical function. Even at diagnostic doses (50 μGy) and therapeutic doses (70 Gy), ionizing radiation alters collagen’s Young’s modulus, indicating internal cross-linking modifications without protein denaturation [7]. Additionally, low-dose radiation (0.5–1 Gy) functionally modulates inflammatory processes through discontinuous dose–response relationships, affecting endothelial cells, immune cell adhesion, and cytokine expression [8]. Therapeutic X-ray doses up to 50 Gy cause extracellular matrix (ECM) alterations and lead to changes in both solubilized and fibrillar forms, leading to significant mechanical alterations, rather than overall morphology [9]. Ionizing radiation modifies the physical interactions between cells and ECM, affecting cell adhesion, migration patterns, mechanotransduction signaling, and differentiation cues [10]. At high doses, ionizing radiation reduces the therapeutic efficacy of transplants such as skin and amniotic tissue grafts due to structural changes in the basement membrane, elastic and collagen fibers, and cytoplasm in epidermal cells [11]. Doses of 10–100 kGy and above lead to cross-linking, cell damage, and death in living tissue, which should be avoided in some cases altogether. Although ionizing radiation is effective for certain applications, it can often cause side effects that limit its use for therapeutic biomaterial modification. For example, exposure of biocompatible materials to ionizing radiation leads to a significant modification of their physicochemical properties by abstraction of hydrogen from α-methyl or methylene groups and generation of free radicals, which affects their clinical use [11]. At the same time, non-ionizing radiation can act as a more controllable and less destructive alternative for tissue modification, allowing for greater spatial and energy precision in the modification of biocompatible tissues, minimizing collateral damage and enhancing recovery processes. Cerenkov luminescence, an intermediate type of radiation, can bridge the gap between ionizing and non-ionizing radiation, overcoming the penetration depth limitation to make photothermal therapies more effective and targeted [12].

Non-ionizing radiation sources can modify tissues in a gentle manner. For example, red and near-infrared irradiation of human tissues is used in clinics [13,14], and cartilaginous tissue modification is used for cell seeding [15]. The treatment of biological tissues with laser irradiation is a well-known strategy for modifying properties and promoting successful healing outcomes [16]. Terahertz irradiation is also used to modify biological tissues [17,18], and to deliver ultrashort, high-voltage pulses [19,20]. With frequencies below approximately 6 THz, the interaction can be understood as a classical electromagnetic wave interaction using the parameters of permittivity and conductivity. At higher frequencies, however, transitions between different molecular vibrational and rotational energy levels become increasingly important, and a quantum mechanical framework is necessary for understanding them [21]. Recently discovered low-energy radiation, such as phonons and excitons, could disseminate in water-containing tissues over the long term. The novel idea is that the combination of various types of bosons could lead to nonlinear synergy in the modification of tissue properties.

Ionizing and non-ionizing radiation both separately lead to energy transfer, but the interactions between the effects of different radiation types could lead to nonlinear effects in affected tissues. One of the nonlinear effects of laser irradiation could lead to self-focusing in living tissues to penetrate deep into scattering tissues for imaging, therapy, or manipulation [22]. This “self-focusing” can be achieved by adjusting wavefronts using techniques like time-reversed ultrasonically encoded optical focusing [23]. Another approach includes the use of the inner properties of cellular biomolecules that can serve as a “guide star” to assist the use of time-reversed light, creating a highly focused beam on the target cells [24], and other techniques involving the interaction effects of electromagnetic fields with living tissues [25,26].

This review aims to investigate the synergistic potential of combining various types of high-density non-ionizing irradiation, an approach we term “bosonic concentrate,” to modify the physiological relevance and functional properties of biological tissues for advanced applications in tissue regeneration and engineering.

## 2. Physiological Requirements for Modification of Tissue Properties

The phenomenon of physiological relevance is the basic concept in tissue engineering, which is responsible for cellular reactions for cellular microenvironment and external effects [27,28], both for native grafts and synthetic materials [29,30]. The lack of physiological relevance of tissue-engineered grafts or transplants not only alters the regulation of resident cells but also leads to immune responses and the development of inflammation.

**Figure 1:**
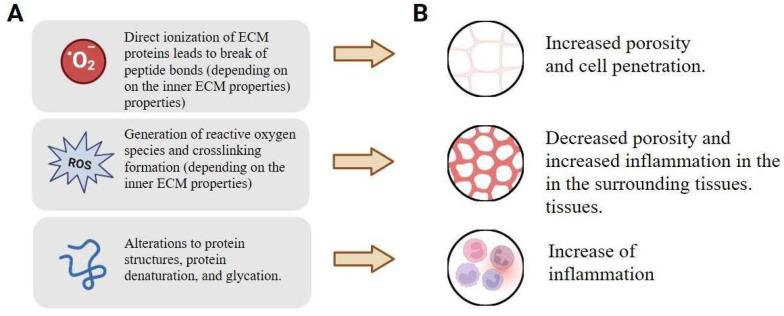
The basics of modifying the physiological relevance of living tissues: (**A**) physical modification of the tissues targets key ECM components like collagen cross-linking and elastic fibers, which directly influence biomechanical properties (e.g., stiffness, elasticity) and cellular mechanotransduction, and (**B**) associated physiological responses include changes in immune cell recruitment and polarization (e.g., macrophages), inflammatory cytokine profiles, and ultimately, the outcome of tissue integration, repair, or fibrosis. Created with Biorender.com.

One of the terms of biocompatibility states that the goal of tissue engineering is to achieve the property of not causing any opposing tissue responses [31]. Tissue graft modifications and insufficient improvement of the synthetic materials lead to a wide range of immune responses released by the acute inflammation with the participation of macrophages and lymphocytes, and cellular inflammation leads to atypical regulation and even delayed cellular reactions based on the cell–cell communications [32,33]. Recent research reveals that biomaterial physicochemical properties, including size, shape, and chemical functionality, directly influence immune activation pathways and can polarize immune cells toward either inflammatory or wound-healing phenotypes [34]. Rather than simply suppressing immune responses, current strategies focus on modulating them to promote tissue regeneration through material design modifications, anti-inflammatory cytokine delivery, and immune cell recruitment approaches [35,36]. This overall approach to understanding the manifestation of immune responses is especially valuable for tissue engineering and regenerative medicine, where detailed investigation and long-term observations are often not feasible. The landscape of the available techniques to modify the tissue properties is presented in Figure 1, which summarizes the two interconnected aspects: the physical modification of tissue properties (Figure 1A), and the subsequent associated physiological responses (Figure 1B).

To rationally design physical interventions, it is crucial to understand how specific tissue conditions can be targeted by specific energy-matter interactions. The differences between various tissues are primarily regulated by the ECM’s biomechanical and biochemical properties, particularly its antigenic properties and regulation of chemotaxis. Rather than merely serving as a passive scaffold, the ECM actively modulates cellular behavior through its biophysical and biochemical properties [37]. The mechanical properties can be quantified and modified. For instance, the mechanical properties of a tissue are largely dictated by its collagen and elastic fiber networks. These properties can be directly influenced by energy inputs. Phononic energy (ultrasound) can induce micromechanical strains that alter fibroblast activity and collagen synthesis, while photonic energy (laser) can induce controlled thermal effects that modify collagen cross-linking density and fiber organization [38].

One of the key parameters is collagen cross-linking, which largely determines the mechanical properties of tissues and cellular interactions. Collagen molecules are stabilized in fibrils by covalent intermolecular cross-links, which are essential for normal tissue physiology and provide tensile strength and viscoelastic properties [39]. Furthermore, the density and type of cross-links directly influence fibril mechanics, determining stiffness under large deformations, as well as failure strain and strength. These cross-links create an interconnected fibrillar material with adjustable impact strength and strength [40]. Cross-linking also significantly influences cellular behavior, manifested by fibroblast-mediated contraction of collagen gels, regardless of cell density [41]. In tissue engineering, cross-linking increases the strength, rigidity, and stability of the construct; however, it can also affect cell viability, adhesion, and proliferation [42]. This presents a key target for energy-based modification. Nonlinear optical effects, such as multiphoton absorption from focused laser irradiation, can precisely induce cross-linking deep within a tissue or scaffold without damaging the surface, thereby enhancing mechanical strength in a spatially controlled manner [43].

The biomechanical characteristics of the ECM depend on the condition of elastic fibers. These fibers determine tissue compliance and are capable of releasing stored energy for passive traction. They also allow tissues to maintain low stiffness and high reversible extensibility [44,45]. Consequently, the degradation of elastic fibers due to aging, pathogenic factors, and hereditary factors leads to tissue dysfunction and provokes systemic diseases, inflammatory reactions, and abnormal physiological changes in tissue [46]. For instance, during the aging process, elastic fibers sustain damage due to enzymatic degradation, oxidative stress, glycation, calcification, and mechanical fatigue. This results in the release of elastin-derived peptides that act as inflammatory mediators, activating cellular processes such as migration, proliferation, and calcification via elastin receptor signaling complexes [47,48]. Therefore, their preservation is critical for maintaining tissue homeostasis and preventing age-related diseases. Energy-based strategies can address this. Low-level laser therapy (photobiomodulation) has been shown to reduce oxidative stress and inflammation [49], potentially mitigating the degradation of elastic fibers [50]. Furthermore, the mechanical energy from acoustic phonons (therapeutic ultrasound) could stimulate the production and organization of elastin by resident cells, promoting tissue compliance. The dynamic proton concentration gradient and oxidative stress in the tissue also influence the matrix’s biomechanical properties. This is primarily due to modulation of critical signaling pathways that cause inflammation, metabolic homeostasis changes, impaired mitochondrial function, and autophagy regulation. These factors contribute to the progression of inflammatory processes and disease [51,52]. In addition, extracellular acidification, caused by ischemic and inflammatory conditions, modulates pro-inflammatory and anti-inflammatory responses, including cyclooxygenase-2 expression and cytokine production via G protein-coupled proton-sensitive receptors [53,54]. Changes in intracellular pH modulate enzyme activity by affecting the ionization state of acidic or basic amino acid residues, disrupting the ionic bonds that determine enzyme conformation, altering substrate binding properties, and regulating cell proliferation, migration, and transformation [55]. Consequently, the regulation of energy metabolism by means of, for example, multi-target drugs that affect several metabolic processes will ensure the preservation of the biomechanical properties of the extracellular matrix of tissues under conditions of possible oxidative stress and ensure the proper quality of tissue engineering products. This metabolic and inflammatory milieu is a prime target for energy-based modulation. Photobiomodulation with red and near-infrared light is known to improve mitochondrial function, shift the cellular redox state towards reduction, and reduce pro-inflammatory cytokine production [56]. By altering the metabolic state of cells, this photonic energy can indirectly normalize the tissue pH and reduce oxidative damage, creating a more conducive environment for repair.

The vast diversity of ECM proteins provides various biochemical and biophysical properties that influence cell phenotype [57]. At the same time, cell composition and density determine tissue metabolic and secretory profiles. For example, cancer-associated fibroblasts (CAFs) regulate cancer cell metabolism through paracrine transfer of metabolites and non-autonomous cell–cell signaling pathways [58]. Another study showed that hepatocyte morphology and liver-specific functions are controlled by varying fibroblast density. Moreover, the different densities of normal human diploid fibroblasts affect the performance of primary rat hepatocytes [59]. Higher cell density increases matrix stiffness, promotes cell clustering, and alters cell patterns that are closely correlated with changes in extracellular matrix elasticity [60]. These cellular patterns and densities can be influenced by energy. Specific wavelengths of light (photons) can guide cell migration and proliferation, a process known as phototaxis, while acoustic patterning with phonons can be used to organize cells into specific architectures within a hydrogel or scaffold, thereby directing tissue formation [61,62].

Cell–matrix mechanotransduction plays an important role in changing the mechanical properties of the matrix. In this process, integrin-mediated and mechanosensitive pathways play a special role [63]. Pattern recognition receptors, including integrins, toll-like receptors, and scavenger receptors, detect and respond to environmental signals [64,65]. In particular, integrins serve as bidirectional biomechanical sensors that transmit signals between cells and the environment. At the same time, their biochemical and mechanical properties ensure specificity in determining the stiffness, composition, and spatial distribution of ECM [66,67]. Therefore, modulation of mechanotransduction pathways allows control of the fundamental processes of tissue development, homeostasis, and disease progression [68]. This provides a direct link for energy-based intervention. The mechanical force exerted by acoustic radiation force from ultrasound (phonons) can directly activate mechanosensitive ion channels and integrin signaling [69,70]. Similarly, photothermal effects can cause localized, transient changes in ECM stiffness, which are sensed by cells, thereby guiding their differentiation or migration.

Another important factor determining the biomechanical properties of the ECM is the regulation of interactions between immune components and the ECM. Immune cells mediate both protective and damaging effects, and their phenotypes and functions depend largely on microenvironmental signals [71]. In particular, macrophages play a crucial role in determining the outcome of tissue remodeling after injury or biomaterial implantation [72,73]. The predominant macrophage phenotype directly influences whether tissue undergoes constructive remodeling or pathological scarring [74]. Macrophage dysfunction, including uncontrolled production of inflammatory mediators or insufficient formation of anti-inflammatory macrophages, contributes to the development of persistent damage and pathological fibrosis [73,75]. Therefore, the degree and phenotype of infiltrating immune cells directly indicate the body’s response and determine subsequent tissue changes. This immune response is highly susceptible to energy-based modulation. As previously mentioned, photobiomodulation can polarize macrophages towards a regenerative, anti-inflammatory (M2) phenotype [76]. Additionally, low-intensity ultrasound (phonons) has been shown to modulate inflammatory signaling pathways, reducing the expression of pro-inflammatory cytokines and promoting a tissue-reparative environment [56,76].

Strategies to modify the physiological relevance of tissues primarily include biochemical interventions, invasive methods, or irradiation of both ionizing and non-ionizing sources, such as laser or ultrasound sources [77]. However, the range and scale of the effects are limited enough that they primarily affect superficial tissues. This requires the use of endoscopic techniques or pharmacological interventions, which are risky for cancer and chronic inflammatory conditions. One possible solution is to use the selected tissue-specific spatial absorption enhancement to increase the depth of radiation penetration into tissues; these effects could be achieved through the nonlinear interaction between radiation and matter.

## 3. Current Applications of Bosonic Concentrate Principles in Biology and Medicine

The concept of “bosonic concentrate” involves the use of various combinations of boson particles (photons, phonons, excitons, and plasmons) to modify biological tissues in order to improve their physiological significance. The use of various combinations of bosons and their unique properties to achieve a synergistic effect allows for high precision and controllability of biological tissue modification processes. This, in turn, leads to controlled changes in the ECM, cell activity, and signaling pathways.

It is crucial to distinguish between the direct, often coherent effects of high-density bosonic concentrates and indirect biochemical pathways, such as the intense production of reactive oxygen species (ROS). While ionizing radiation and some high-intensity photodynamic therapies act primarily through stochastic damage, including DNA breaks and significant ROS formation that overwhelms cellular defenses, the “bosonic concentrate” approach targets a controlled energy deposition below the damage threshold. The main interactions we focus on include coherent energy transfer (e.g., Förster resonance energy transfer—FRET, exciton migration) for precise molecular targeting; localized thermal effects from photon–phonon interactions for modifying ECM properties; mechanical forces and strains from acoustic phonons for stimulating cellular mechanotransduction; and enhanced optical phenomena from excitons for signal amplification and hyperlocalized heating.

In this paradigm, ROS production is typically a potential secondary, and often undesirable, outcome that must be managed or minimized, rather than the primary intended mechanism of action. The goal is to use the collective properties of bosons to “tune” the tissue’s physical state with high precision, moving beyond causing indiscriminate oxidative stress.

### 3.1. Phenomenon of Bosonic Concentrate in the Condensed Matter

Bosons, such as photons or phonons, are particles that mediate forces and can occupy the same quantum state. This property allows them to behave collectively and coherently. In medicine, this property enables photons to align in phase and energy within a laser cavity, producing a highly focused and powerful beam of light, from delicate surgical cutting to targeted tissue therapy [78,79]. Phonons, which are quantized vibrations in tissues, interact by scattering and absorbing ultrasound waves, and these interactions influence diagnostic information in medical imaging [80,81,82,83].

The “bosonic concentrate” is a therapeutic or diagnostic method that uses multiple bosonic particle types (e.g., photons, phonons) in simultaneous or sequential applications to maximize their combined effects on biological matter. Photodynamic therapy and laser tissue modification can be performed using gamma quanta and visible light photons with characteristic energy ranging from 0.1 eV to 10 MeV. Acoustic phonons, which are quanta of sound, have characteristic energy of 0.03–0.1 eV and are used in ultrasonic therapy to stimulate tissue regeneration and improve drug delivery. Excitons, which are excited states formed by the interaction of electrons and holes, have characteristic energy around 0.1 eV and can be used to mediate the biochemical reactions within cells. Plasmons, which have characteristic energy of approximately 1–10 eV and are quasiparticles that arise from the collective oscillations of an electron gas under high-frequency electromagnetic radiation, can be applied to enhance the efficiency of photothermal therapy and amplify signals in biosensors. A physical characteristic of bosons is their ability to condense to the lowest energy levels, ultimately leading to condensation [84]. Biological tissue-specific radiation is referred to as “biophotons,” denoting the permanent, spontaneous emission of photons by all living systems within the spectral range of at least 260 to 800 nm. Biophotons originating from spontaneous or light-induced living systems display super-Poissonian, Poissonian, and sub-Poissonian statistical distributions. This finding provides the first evidence of non-classical light in living tissue [85].

Thus, “bosonic concentrate” is a term that refers to a combination of radiation that can modify the ECM’s properties, thereby changing the material’s structural and functional properties at the molecular level. Coherent energy transfer, involving synchronized movement of energy between molecules or particles, plays a crucial role in living tissues by facilitating efficient energy distribution, affecting cellular regulation in vivo [86]. In bosonic interactions, the collective action principle refers to the phenomenon of quantum composition, in which multiple bosons act together to produce effects that are greater than the sum of their individual actions.

### 3.2. Photons: Laser and Maser Sources

In living tissues, photons are absorbed, emitted, or scattered, leading to biological effects essential for processes like vision, photosynthesis, and phototherapy. Similarly, phonons, quanta of vibrational energy, can contribute to the mechanical properties of cells and tissues by participating in various vibrational modes [87].

Far-infrared (FIR) radiation uses wavelengths ranging from 15 μm to 1 mm. Compared to visible light and near-infrared radiation, FIR photons have lower energy, making them less likely to cause direct damage to biological tissues. Instead, FIR is often used for its thermal effects, which can improve blood flow, reduce muscle stiffness, and promote relaxation. Near-infrared (NIR) radiation uses wavelengths ranging from 700 to 1400 nm. NIR photons can penetrate deeper into biological tissues than visible light can, making NIR particularly useful for medical imaging and therapeutic applications. NIR irradiation is used in techniques such as photobiomodulation, which stimulates cellular processes, enhances tissue repair, and reduces inflammation [88].

Terahertz (THz) radiation occupies the electromagnetic spectrum between microwaves and infrared light. It typically falls within the 0.1 to 10 THz range. Although THz radiation is non-ionizing and generally considered safe for biological tissues, it can interact with water molecules and lead to localized heating. THz imaging is an emerging, powerful tool for noninvasive diagnostics [89]. THz irradiation can provide detailed images of tissue structures and identify abnormalities, such as tumors [90].

Masers operate in the microwave portion of the electromagnetic spectrum. Like lasers, they emit photons, but instead of visible or infrared light, they emit microwave photons. Although masers are less commonly used in biological applications than lasers, their unique properties make them valuable in certain contexts. For example, masers can be used for highly sensitive magnetic resonance imaging and spectroscopy, which provides detailed insights into the molecular composition and structure of tissues [91,92].

### 3.3. Acoustic Phonons

The interaction between acoustic phonons and photons in living tissues is a complex phenomenon that can be explained by the principles of acousto-optic effects and Brillouin scattering. The acousto-optic effect involves modulating light with sound waves. As acoustic phonons propagate through tissue, they can create periodic variations in its density and refractive index [93].

Brillouin scattering is a specific type of light scattering that occurs when photons interact with acoustic phonons. During this process, the incident photons are scattered by density fluctuations caused by acoustic phonons. This results in a shift in the frequency of the scattered light. One advantage of Brillouin scattering in biological tissues is that associated acoustic phonons scatter much less than photons [94], and acoustic phonons cannot be scattered in tissues like photons [95], allowing for deeper probing of mechanical properties. By analyzing the frequency shift of the scattered light, researchers can obtain detailed information about the biomechanical properties of these tissues. This could lead to improved diagnostic techniques and treatment options [96].

### 3.4. Other Particles and Quasiparticles

The role of excitons in living systems is still unclear, but some hypothesize that they may facilitate the highly efficient energy transfer processes observed in photosynthetic organisms, vision, and the function of certain proteins [97]. According to theoretical models, excitonic interactions could facilitate this energy transfer by enabling coherent energy transport, which could be observed in multiphoton absorbance in the high-intensity coherent radiation [98].

Solitons can also be produced by high-density irradiation as stable, localized wave packets that can travel long distances without changing shape due to a balance between nonlinear and dispersive effects [99]. Other methods of bosonic production are related to stimulated Raman scattering (SRS) and stimulated Brillouin scattering (SBS). In SRS, incident photons are scattered by a medium, resulting in a frequency shift of the light and the generation of new photons [100]. SBS is similar to SRS but involves the interaction of light with acoustic phonons, leading to frequency shifts and amplification of the scattered light [101].

## 4. Nonlinear Interactions and Synergisms of the High-Density Non-Ionizing Irradiation

Nonlinear interactions and synergistic effects in biological tissues are crucial for understanding the complex dynamics arising from the interplay of various physical phenomena, which stem from their molecular complexity [102,103,104]. One such interaction is the thermal effect resulting from photon–phonon interactions within a simple tissue [105]. When photons (quanta of light) interact with phonons (quanta of vibrational energy in a tissue’s lattice structure), energy is transferred, leading to localized heating. This thermal effect can significantly influence tissue properties and behavior, affecting processes such as cellular metabolism, signal transduction, and the efficacy of therapeutic interventions like laser treatments. Resonance energy transfer and multiphoton absorption are common mechanisms by which high-density quantum flow affects biological tissues.

Förster resonance energy transfer (FRET) is a powerful technique used to study interactions between biomolecules on the nanometer scale. It involves the non-radiative transfer of energy from a donor fluorophore to an acceptor fluorophore when the two are in close proximity, typically within 1–10 nm [106,107]. This phenomenon is highly sensitive to the distance and orientation between the donor and acceptor, making FRET an invaluable tool for investigating molecular interactions, conformational changes, and the dynamics of complex biological systems.

Multiphoton absorption is a nonlinear optical process in which a molecule is electronically excited by the simultaneous absorption of two or more photons. This phenomenon is useful for biological imaging and phototherapy because it provides high spatial resolution and deep tissue penetration [108,109]. For instance, multiphoton microscopy allows for visualization of intricate structures within living tissues with minimal photodamage and photobleaching.

A summary of the possible effects caused by the synergistic application of non-ionizing radiation due to the heterogeneity of living tissue properties is presented in Table 1.

The interaction between bosonic concentrate releases and biological tissues appears to be significantly impacted by the intrinsic heterogeneity of the ECM and its associated biochemical environments. Variations in ECM organization, including the spatial orientation and linearity of fiber structures, can modulate optical responses by generating density- and direction-dependent patterns. These patterns affect the induced optical image of the tissue [104,110,111]. Similarly, changes in ECM density alter the balance between macromolecular content and tissue hydration. Reduced ECM density increases water content, which can lead to local overheating and reduced radiation penetration [107,112,113,114]. Additionally, the presence of specific metabolites, particularly electron donors, is suggested to alter radiation scattering properties and influence localized photon distribution within the tissue matrix [115,116]. These microstructural and chemical variations collectively shape the magnitude and localization of bosonic effects, determining the efficiency and selectivity of photonic interactions at the tissue level.

Beyond the extracellular environment, cellular and metabolic characteristics further enhance the complexity of tissue responses to bosonic concentrates. Alterations in cellular ultrastructure, including modifications to membrane lipid composition, organelle density, and cytoskeletal organization, may promote increased multiphoton absorption and FRET efficiency. This amplifies nonlinear photonic signaling and induces stress-related cellular responses [115,117,118]. Metabolic heterogeneity, exemplified by fluctuations in ATP concentration, enzymatic activity, and redox potential, can influence photon–phonon thermal interactions. This can lead to uneven tissue heating and metabolism-dependent variations in resonant energy transfer [56,119,120,121]. Concurrently, tissue oxygenation gradients, which arise from uneven perfusion or localized hypoxia, modify scattering and absorption characteristics. This affects the extent of oxidative stress and the penetration depth of radiation [114,122,123,124]. These phenomena may be particularly pronounced in neural tissues, where shifts in synaptic density and neurotransmitter profiles facilitate heightened nonlinear signal propagation through FRET and multiphoton absorption mechanisms. This effect modulates neurosignaling pathways and may contribute to excitotoxic effects [56,126,127].

## 5. Application of Combined Non-Ionizing Irradiation In Vivo

### Combined Effects of Irradiation on Biological Tissues

Currently, essential data on the effects of ionizing and non-ionizing radiation on biological tissues is lacking. Laser irradiation with infrared and visible light allows for the ablation of collagenous material and leads to pore formation [128,129,130]. However, not only are biological macromolecules modified, but the process could also affect immune cells and their responses to material [129]. Indeed, red irradiation directly into blood vessels decreases inflammation [131,132], primarily due to immune cell regulation modification.

**Figure 2:**
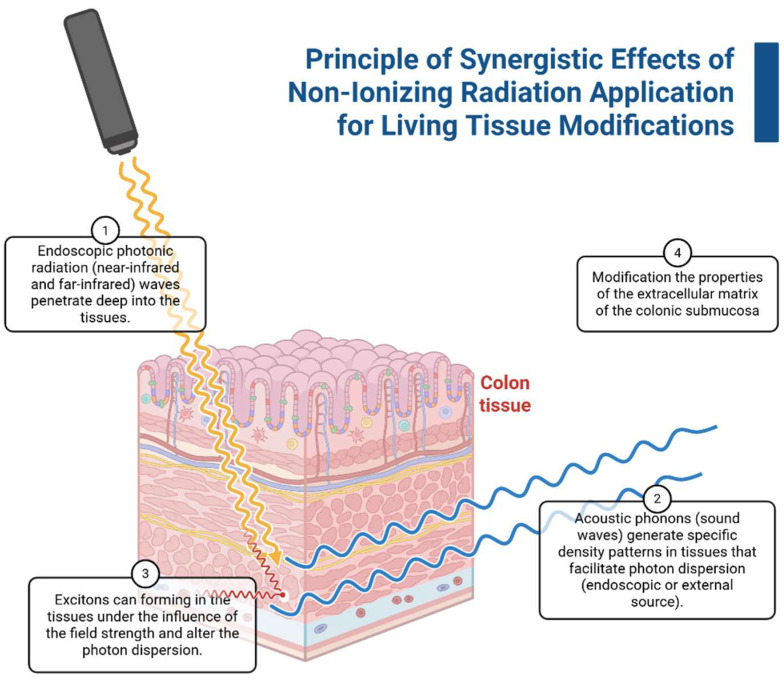
Principle of combined non-ionizing irradiation for living tissue modifications (using colon tissue as an example). Created with Biorender.com.

The application of the bosonic concentrate principle is presented in Figure 2. Initial infrared radiation penetrates the colon tissue, and density patterns form simultaneously with the phonon source; biochemical-dependent electronic emission or exciton pair formation leads to cross-linking of macromolecules.

Therefore, single high-density radiation sources could be used for therapeutic purposes. A combination of high-yield, non-ionizing radiation sources could form a three-dimensional lattice structure for wave scattering or diffraction and modify the spatial properties of the scaffolds with greater precision and less collateral damage.

The physiological relevance of scaffolds and the requirements for their modification play an important role in current biotechnology. Physiological compatibility refers to the harmonious interaction between different biological tissues or implants. This ensures that they function together effectively without causing adverse reactions or disruptions. This concept is crucial in medicine, pharmacology, and biotechnology because it ensures that introduced substances, devices, or organisms do not negatively impact the host’s normal physiological processes. Physiological compatibility is commonly described in terms of elastic modulus and tensile strength, which are related to mechanical trauma in implants or related tissues [29,133,134]. Development of trauma can lead to local inflammation, release of pro-inflammatory factors, and formation and ingrowth of fibrous tissue.

The role of tissue inflammation in the response to radiation is critical for the reactions of immune cells, primary mast cells, and macrophages [135]. In fact, small changes in the macromolecular organization of the scaffold, which required precision techniques, caused surface modification and changes in cell adhesion. These changes will modify the immune response. Immunological methods are indeed the most informative for assessing the biological properties of implants [129,136].The radiation techniques application leads to modifying the biological tissue properties [30,137,138], not only the spatial organization but also the physiological relevance and cellular mechanotransduction, which in turn can alter the biocompatibility of biomaterials [132,139,140].

Differential properties of normal and tumor tissues involve morphological, immunological, and topological biomechanical properties. The topological features of tissues can be exploited to study the heterogeneous effects of radiation on tissues [141]. For instance, the predominantly linear arrangement of collagen filaments in tumor tissues can facilitate specific boson scattering [142]. Modifying the tumor microenvironment and reverting tumor cells will be a novel strategy for treating malignant tumors [143], especially as premedication for solid tumors [143,144].

The combined application of various types of high-density, non-ionizing radiation in living tissues is marked by nonlinear optics and radiophysics and can be summarized as the phenomenon of “bosonic concentrate.” This phenomenon could be applied not only to therapy but also to diagnostics because forming tissue-specific patterns could enhance the resolving power of diagnostic devices by serving as specific reflection or dispersion markers (spectral biomarkers) [145].

## 6. Limitations and Future Directions

The principles underlying bosonic concentrate applications are founded on the diffraction of light by sound in solids [146]. Currently, this effect is primarily used in electronics, and only a few studies have investigated its effects on living tissues. The bosonic concentrate concept differs from single high-irradiation due to the interface’s impact on the physical properties of biological tissues and their variations in normal, damaged, and tumorous tissues. Indeed, resolving the optical effects on biological tissues requires energy, which can lead to tissue damage [4,147], and requires treatment options [148]. This issue could be solved by choosing the relevant frequency and using enhancer agents, as in Raman spectroscopy. Another limitation of this study was that it did not perform calculations of the tissues’ physical properties to verify the feasibility of the described effects using currently available techniques.

In addition, there are two hypothetical features that raise critical, fundamental objections to the feasibility of this approach. The first relates to the irregularity and lack of ordered structures in the extracellular and cellular environments of living tissues, which distinguishes them from chaotically organized materials. The second is that such regular structures do exist; however, their interference precludes any use of nonlinear effects. Currently available data does not allow us to determine the accuracy of any of these features.

The promised application of the integration of different types of radiation lies in the field of low-wavelength, high-intensity photon emission, which can penetrate deep into living tissues and disperse across temporarily phonon-formed regular structures. This process not only produces biophotonic effects in affected tissues but also enables efficient energy transfer for the physical modification of tissue. Due to differences in tumor and healthy tissue properties, dispersion and energy transfer depend on both the variations in optical and mechanical properties and the biochemical characteristics and molecular complexity of the different types of tissues [149] and could be used for cancer treatment. Currently, the interaction of different types of radiation could be presented as extended space dimensions, which use the advanced quantum electrodynamic models [150], and also could be visualized as 4D models for tissue engineering and microfabrication [151].

The recently published studies showed that interactions between different types of radiation can yield emergent effects, including synergistic and antagonistic responses [152]. The simplest synergistic effects of the complicated interaction between ionizing and UVB radiations in normal human cells are manifested by persistent DNA damage, altered stress gene expression, and increased chromosomal instability, which exceeds the pure additive action of the radiations in most cases [153]. However, the nonlinear interaction leads not only to direct cell damage but also to the imaging of functional and molecular information in living tissues [154]. For example, advances in hybrid optical-optoacoustic microscopy offer a next step in multimodal interrogations [155], opening novel capacities of the biochemical-adjuvanted photoacoustic methods in diagnostics and therapy.

## 7. Conclusions

High precision and control in the process of modifying biological tissues allow for minimized damage to surrounding healthy tissues and improved recovery processes. Nonlinear optics currently presents potential treatment options for modifying tissue-specific compatibility in biological tissues based on various combinations of synergistic effects. These options are based on key non-ionizing radiation effects according to dominant energy transfer mechanisms: (1) transverse vibrational processes arising from phonon interactions with biological tissue and (2) energy transfer from electromagnetic fields to materials associated with photon and exciton absorption. Using these techniques can contribute to creating new biomaterials with specific biological properties, which is important for developing artificial implants, efficient drug delivery systems, and tissue engineering approaches.

## Figures and Tables

**Table 1:** Possible effects caused by the bosonic concentrate applications due to heterogeneity of the intrinsic properties of living tissues.

Factor	Source of Differences	Effect	Refs
ECM organization	The presence of spatial patterns, preferred direction and linear organization of fibers.	Stimulating a specific optical image and density pattern.	[104,110,111]
ECM density	Reduced density leads to a reduction in the proportion of macromolecules and increases hydration.	Increased water content leads to local overheating and reduces the radiation penetration.	[112,113,114]
Specific metabolites presence	Excess presence of electron donors.	Specific effects of radiation scattering.	[106,115,116]
Cellular Structure	Changes in the structure and composition of membrane lipids, organelle density, and cytoskeletal organization.	Increased multiphoton absorption and FRET efficiency, leading to enhanced nonlinear signaling and potential cellular stress responses.	[115,116,117,118]
Metabolic States	Significant differences in ATP levels, enzyme activity and redox potential in different tissues.	Modulation of photon–phonon thermal effects, causing uneven heating and changes in metabolic rate, synergistically combined with resonant energy transfer for targeted biomodulation.	[119,120,121]
Tissue oxygenation	Heterogeneous oxygen gradients caused by perfusion and hypoxia in various areas.	Influence on the scattering and absorption of boson concentrates, leading to local oxidative stress or increased penetration into hypoxic areas.	[122,123,124]
Neural connections	Changes in synaptic density and neurotransmitter profiles in neural tissue.	Enhancement of nonlinear signal transmission via FRET and multiphoton absorption, leading to altered neuromodulation or excitotoxicity.	[56,125,126,127]

## Data Availability

No new data were created or analyzed in this study. Data sharing is not applicable to this article.

## Abbreviations

ECM	Extracellular Matrix
FIR	Far-infrared radiation
NIR	Near-infrared radiation
SBS	Stimulated Brillouin Scattering
SRS	Stimulated Raman Scattering
